# Managing the Little Ones

**Published:** 1891-04

**Authors:** 


### MANAGING THE LITTLE ONES.

Babies grow so fast; they change almost from day to day, and the little trick that you think not quite pretty will be forgotten to-morrow or a day later, if you say nothing to the baby about it, but induce him to stop it simply, by attracting his attention to something else. The whole secret of "training" baby is to furnish him amusement, or let him find it for himself; and in case he finds something amusing that it is best and wise he should not have, draw his attention to something else, and quietly let the other attraction disappear. Don't imagine he will think you are afraid to correct him; he has not the least idea on the matter at all, and does not realize that he is "managed;" he simply knows he is having a good time. By and by, when baby is old enough to understand causes and effects, and you first explain your reasons to him, *then* if he does not obey it may sometimes be necessary to slap a hand ; for as soon as a child is old enough to understand what you tell him, and the reason why, he must not be allowed to realize that he can disregard the parents' commands. Make as few rules as possible. Never make a rule or give a command unless it is necessary and wise to do so ; but when done, then gently but firmly enforce it. On the other hand, give praise and kisses for prompt obedience. Let the child overhear you telling papa that he is just a splendid boy to obey ; that you always know when you tell him not to do a thing that he will obey you, and slyly watch the effect. It will please the little one and help it to obey in future. Never rule by "don'ts." Praise the little ones all you can truthfully, and find fault as seldom as possible.

### INJURIOUS MIXTURES.

The iodide of potassium is quite a constant ingredient of sarsaparilla mixtures, which are vaunted to be "blood purifiers," tonics, etc. This agent is harmless when rightly used, but it is capable of doing grievous injury. One of its baneful effects is inflammation of the kidneys. If they are weak or deranged, small doses of the iodide are likely to produce the effect stated ; and many persons' kidneys are so affected without the fact being known. Hence, preparations of the class mentioned should be held as unsafe for general use. Besides this, they are, as a rule, absolutely worthless, even in the most carefully selected cases.

